# The Relation Between Perfusion Pattern of Hepatic Artery Perfusion Scintigraphy and Response to Y-90 Microsphere Therapy

**DOI:** 10.4274/Mirt.77487

**Published:** 2013-12-10

**Authors:** Bilge Volkan-Salancı, Murat Fani Bozkurt, Bora Peynircioğlu, Barbaros Çil, Ömer Uğur

**Affiliations:** 1 Department of Nuclear Medicine, Hacettepe University, Medical Faculty, Ankara, Turkey; 2 Department of Radiology, Hacettepe University, Medical Faculty, Ankara, Turkey

**Keywords:** Yttrium radioisotopes, microspheres, liver neoplasms, hepatic artery, Radionuclide imaging, Perfusion

## Abstract

**Objective:** Hepatic artery perfusion scintigraphy is a routine procedure for patient evaluation before Y-90 radiomicrosphere therapy and mostly used for prediction of extrahepatic leakage. Moreover, it also displays perfusion pattern of tumours, which is an important parameter on success of the therapy. The aim of this study is to assess the relation between the perfusion pattern on hepatic artery perfusion scintigraphy and radiomicrosphere therapy response.

**Methods: **A total of 99 radiomicrosphere therapy applications were carried out in 80 patients (M/F: 55/25).

**Results: **Heterogeneous and diffuse perfusion patterns were observed in 47 patients and 52 patients, respectively. The patients with diffuse perfusion pattern had better therapy response both on FDG PET/CT (p= 0.04) and CT (p=0.008) when compared to those with heterogenous perfusion pattern.

**Conclusion:** Perfusion pattern observed on hepatic artery perfusion scintigraphy may be a successful predictor of early response to radiomicrosphere therapy.

**Conflict of interest:**None declared.

## INTRODUCTION

Yttrium-90 radiomicrosphere therapy is used for treatment of unresectable primary or metastatic liver disease. Diagnostic angiography is combined with hepatic artery perfusion scintigraphy (HAPS) to assess extrahepatic microsphere leakage. Presence of pulmonary shunting is quantified and used for therapeutic Y-90 microsphere dose calculation. SPECT/CT images are found to be superior to planar or SPECT imaging for detection of gastrointestinal shunting (1).

Liver tumours’ neovasculature arise from hepatic artery, however their disordered nature may result in heterogeneity of tumour perfusion. Assessment of tumour perfusion is essential in Y-90 microsphere therapy. Although, it is not identical Tc-99m labelled macroaggregated albumin (99mTc- MAA), used in HAPS, has similar particle size with Y-90 microspheres and is believed to simulate Y-90 microsphere distribution (2). The amount of Y-90 activity injected and the absorbed radiation dose also depends on the distribution of microspheres, which results from the variable hemodynamic conditions within the hepatic artery tree and the vessel density inside the tumours (3). Therefore, hepatic tumours with better perfusion are expected to give better response to Y-90 microsphere therapy. In this study, the relation between perfusion pattern on HAPS and radiomicrosphere therapy response was assessed. The tumour perfusion pattern was determined visually using HAPS SPECT images and the therapy response was assessed by F-18 FDG PET/CT and abdominal CT. 

## MATERIALS AND METHODS

The HAPS of 80 patients, who received Y-90 resin microsphere therapy (SIR-Spheres, SIRTEX) between April 2008 and July 2011, were retrospectively analyzed. All of the patients had the diagnosis of either primary hepatocellular cancer (HCC) or unresectable liver metastasis. The hepatic function reserve, performance scores and tumour stage were evaluated prior to the therapy. All patients underwent at least one diagnostic imaging test (abdominal CT, MRI and/or FDG PET/CT) at baseline.

HAPS was performed in all patients following diagnostic angiography. A dose of 185 MBq 99mTc-MAA (Pulmocis, IBA molecular) was prepared just before the diagnostic angiography and injected to the hepatic artery or one of its branches accordingly. Whole body and SPECT imaging was carried out within 1 hour following the tracer administration on a dual-headed gamma camera equipped with low-energy ultra high resolution collimators (Siemens ECAM, Erlangen Germany). SPECT acquisition parameters were: peak energy 140 keV (window: 20%), step and shoot protocol, 25 s/projection at 256x256 matrix. Images were reconstructed using filtered back projection algorithm, and Butterworth filter (order:0,5 and cut off:10). SPECT/CT imaging was carried out using hybrid SPECT/CT gamma camera (GE Hawkeye, USA) in case any gastrointestinal shunting was suspected. CT component was acquired by 10 mm axial sampling, 140 kpV, 2.5 mA, and at 256X256 matrix size.

HAPS images were evaluated visually and perfusion pattern of the tumour was classified into 2 categories as heterogeneous or diffuse. Semiquantitative analysis was carried out for pulmonary shunt fraction (PSF) using the following formula:

PSF= lungs’ counts/(liver counts+lungs’ counts)*100.

Administered dose was calculated using the body surface area (BSA) based formula (Dose=BSA-0.2+Tumour/Liver) and the calculated dose was reduced if the pulmonary shunt was higher than 10%. After radiomicrosphere therapy administration, the therapy response was assessed either by CT (performed after one month and after at least 3 months), MRI or FDG PET/CT in 35 patients. Results of anatomic and metabolic imaging were graded using RECIST and PERCIST criterion, respectively.

Statistical significance was accepted when p values were smaller than 0.05. The relation between therapy response and HAPS pattern was analyzed using Chi-square test. The relation between baseline tumour diameter and perfusion pattern was assessed using Mann-Whitney U test. 

## RESULTS

Ninety-nine radiomicrosphere therapy applications were carried out in 80 patients (M/F: 55/25). The median age of the patients was 58 years (32-79). The baseline characteristics and the diagnoses of the patients were listed in Table 1 and 2, respectively. Four patients had left and 2 had right liver lobectomy before radiomicrosphere therapy.

Mean tumour diameter with regard to the largest lesions was 6.4±3.8 cm in HCC and 5.6±4.7 cm in metastatic tumours (p= 0.570) according to baseline.

None of the patients had evidence of gastrointestinal shunting. Mean time elapsed between HAPS and radiomicrosphere therapy was 33 days. Mean PSF calculated in HAPS was 3.0%±1.9 [range: 0.9% - 9.0%], where none of the treated patients had PSF over 10%. Heterogeneous uptake and diffuse uptake of MAA was observed in 47 patients (47.5%) and 52 patients (52.5%), respectively.

The mean Y-90-microsphere dose given to the patients was 1.23±0.22 GBq (range: 0.7-2 GBq). Right lobe was treated in 76 patients (mean administered dose 1.25±0.2 GBq), left lobe in 16 patients (mean administered dose 1.0±0.2 GBq), and bilobar application was done in 7 patients (mean administered dose 1.5±0.3 GBq). Seventeen patients received the therapy twice and three patients received therapy three times. Mean time interval between two radiomicrosphere therapy applications was 165.3±130.7 days.

None of the patients suffered from any complications related to gastrointestinal leakage after radiomicrosphere therapy. Thirty-five patients’ therapy response was evaluated by both abdominal CT and FDG PET/CT. A control PET/CT was performed after 1-3 months in order to evaluate metabolic response to therapy. Three patients with ‘stable disease’ were included in ’progression’ group for statistical analysis. Most of the patients with heterogeneous uptake in HAPS showed partial metabolic response (n=12/15), whereas more than half of the patients with diffuse uptake pattern displayed complete metabolic response (n=11/20) (Table 3).

For therapy response evaluation, the patients’ control CT was performed both after one month (n= 63 pts) and after 3 months (n=35 pts) at minimum. There was no difference in therapy response measured by CT between perfusion patterns after one month. However, in the second follow-up CT carried out after a mean of 4.8±3.6 months; 9/21 (25%) patients had progressive disease in heterogeneous uptake group. The remaining patients were either responders or had stable disease (p= 0.008) as shown in Table 4.

**Figure 1:**
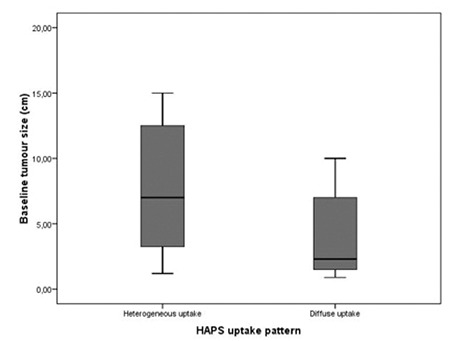
Graph showing baseline tumour size difference between 2 differentHAPS perfusion patterns. Larger tumours have tendency to showheterogeneous perfusion pattern

The tumour size was greater in heterogeneous uptake group when compared to diffuse uptake group (p= 0.004) (Figure 1). Smallest tumour diameter was observed in complete metabolic responders (tumour diameter: 2.5±0.5 cm). In partial metabolic responders and in patients with progressive disease, tumour diameter was 8.6±4.1 cm and 7.4±8.0 cm, respectively. However, this difference did not reach statistical significance (p= 0.058). The radiation doses given to the patients with heterogeneous perfusion pattern in HAPS were significantly higher when compared to those of diffuse uptake group (1.3±0.23 GBq vs. 1.18±0.19 GBq; p= 0.029).

## DISCUSSION

In this retrospective study, the perfusion patterns in 99 HAPS studies of 80 patients were visually grouped as diffuse and heterogeneous uptake. The therapy response was evaluated after SIRT either by CT/MRI or FDG PET/CT and any relation between therapy response and HAPS perfusion patterns was sought.

HAPS routinely combined with diagnostic angiography, is mainly used to evaluate the presence of extrahepatic shunts either to lungs or to gastrointestinal system (4). In this study, PSF was calculated from whole body images. None of the patients had PSF that was higher than 10%. SPECT and SPECT/CT was performed for detection of gastrointestinal leakage. The patients with signs of gastrointestinal leakage was either corrected by interventional radiologist on a repeated angiogram, or referred to an alternative therapy. None of the patients reported here suffered from any complication related to gastrointestinal shunting after therapy. These results indicate that patient preparation was appropriate in study population.

Liver tumours’ nutrient vessels originate from hepatic artery. This tumour neovascularity is essential for survival of the tumour. During diagnostic angiography 99mTc MAA particles are injected through hepatic artery and it is assumed that their distribution pattern can be used for relative density of microvasculature at the time of the injection and MAA particles trapped in arteriolar-capillary bed demonstrates tumour vascularity better than hepatic angiography (5,6). Ho et al. found no correlation between tumour size and vascularity except for tumours smaller than 20 cm. They reported that tumour diameter greater than 20 cm showed reduced vascularity (6). In a study by Flamen et al. MAA distribution was found to be similar to Y-90 resin microspheres. They assessed tumour perfusion semiquantitatively, and revealed that MAA perfusion is a good response predictor with regard to given Y-90 activity, and the simulated absorbed dose (7). In another study, the tumours’ perfusion change was evaluated by HAPS perfusion pattern after a reperfusion procedure i.e. occlusion of extrahepatic vessels, and relation between perfusion pattern change and tumour response was evaluated (8). The authors stated that HAPS revealed the change in perfusion after redistribution during the angiographic workup, and this way they delivered treatment to the whole tumour volume accurately (8). In the present study, the perfusion pattern was assessed visually and a heterogeneous perfusion pattern was observed especially for liver lesions with greater diameter and necrotic centre (Figure 1). Smaller tumours displayed diffuse and intense uptake of 99m-Tc MAA. This finding supports the literature stating that HAPS can be used to evaluate not only the extrahepatic shunting but also tumour vasculature in order to predict therapy response (7,8).

In a study performed on 58 colorectal hepatic metastasis patients HAPS uptake intensity was qualitatively grouped, and tumour response was assessed with CEA levels and CT scans performed after 1 and 2 months (9). The authors reported that the intensity of uptake was not a predictor of tumour response and they did not find any statistical difference in survival, either. However, in that study the tumour response was assessed relatively early and primarily by anatomical imaging (9). In another study performed on colorectal liver metastasis, the degree of MAA uptake was not correlated with tumour response evaluated with MRI after 6 weeks and 3 months (10). However they grouped bigger necrotic tumours with ones with strong uptake. We believe that tumour perfusion is heterogeneous, especially if the tumour is greater in diameter. This heterogeneity might result in inhomogeneous distribution of not only MAA particles but also microspheres. In our opinion, this is why we found a relation between tumour perfusion pattern and metabolic response to therapy.

Factors affecting therapy response after SIRT for HCC was investigated in many studies and intensively reviewed (11). Most of them classified the patients according to staging systems. However in addition, portal vein thrombosis, radiation dose given (>120 Gy or <120 Gy) or number of nodules present in the liver were also evaluated (12,13). The therapy response to SIRT mainly depends on the radiation dose given, tumour hemodynamics and tumour’s vascular density (3). Tumour perfusion was assessed by MRI or dynamic CT in many studies however the relation with therapy response and tumour perfusion was not targeted in most of them. In a recent study, Reiner et al showed that perfusion measured by dynamic CT was similar to 99mTc MAA perfusion (14). In the present study, although qualitatively assessed, the perfusion pattern predicted the therapy response on the basis of both CT and FDG-PET/CT evaluation. A better therapy response was noted for tumours that had diffuse perfusion pattern in HAPS.

In conclusion HAPS can display not only the extrahepatic leakage but also tumour perfusion pattern, therefore may be used for evaluation of therapy response or Y-90 microsphere dose calculation. 

## Figures and Tables

**Table 1:**
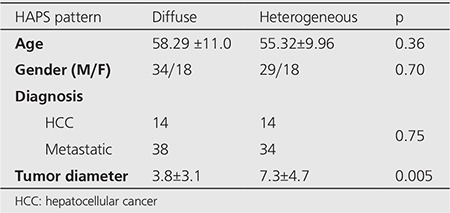
Baseline characteristics of the patients

**Table 2:**
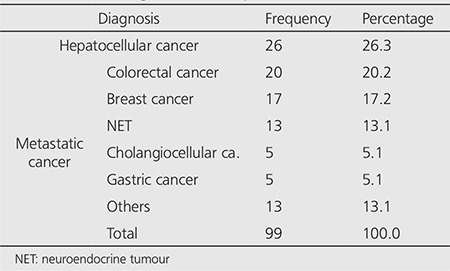
The diagnosis of the patients

**Table 3:**
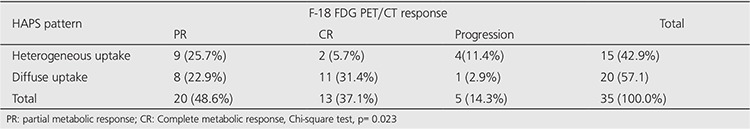
The relation between HAPS pattern and metabolic response

**Table 4:**
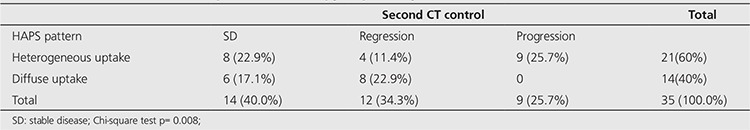
Relation between HAPS pattern and therapy response by CT after 3 months
